# Re-wiring the brain: Increased functional connectivity within primary somatosensory cortex following synchronous co-activation

**DOI:** 10.1016/j.neuroimage.2014.01.052

**Published:** 2014-05-15

**Authors:** Rishma Vidyasagar, Stephen E. Folger, Laura M. Parkes

**Affiliations:** a Centre for Imaging Sciences, Institute of Population Health, University of Manchester, M13 9PT, UK; b Magnetic Resonance Imaging and Analysis Research Centre (MARIARC), University of Liverpool, L69 3GE, UK; c Department of Physical Therapy Education, Elon University, Campus Box 2085, Elon, NC 27244-2085, USA

**Keywords:** Coherence, Cortical reorganisation, fMRI, Plasticity, BOLD, Somatotopy

## Abstract

The primary somatosensory cortex shows precise topographical organisation, but can be quickly modified by alterations to sensory inputs. Temporally correlated sensory inputs to the digits can result in the merging of digit representations on the cortical surface. Underlying mechanisms driving these changes are unclear but the strengthening of intra-cortical synaptic connections via Hebbian mechanisms has been suggested. We use fMRI measures of temporal coherence to infer alterations in the relative strength of neuronal connections between digit regions 2 and 4 following 3 hours of synchronous and asynchronous co-activation. Following synchronous co-activation we find a 20% increase in temporal coherence of the fMRI signal (p = 0.0004). No significant change is seen following asynchronous co-activation suggesting that temporal coincidence between the two digit inputs during co-activation is driving this coherence change. In line with previous work we also find a trend towards reduced separation of the digit representations following synchronous co-activation and significantly increased separation for the asynchronous case. Increased coherence is significantly correlated with reduced digit separation for the synchronous case. This study shows that passive synchronous stimulation to the digits strengthens the underlying cortical connections between the digit regions in only a few hours, and that this mechanism may be related to topographical re-organisation.

## Highlights

• Synchronous and asynchronous co-activation paradigm on digits 2 and 4 in humans
• Increase in BOLD temporal coherence between both digits in the synchronous case
• Suggests increased functional connectivity in the somatosensory region
• Increased coherence correlates with decreased digit separation
• Coherence changes suggests Hebbian mechanisms may be responsible.

## Introduction

The dynamic nature of neuronal circuitry allows the brain to change and develop in response to external stimuli. These changes in synaptic processing can ultimately lead to topographical reorganisation of cortical regions (Merzenich et al., 1983). One of the mechanisms that has been shown to steer these changes follows the Hebbian rule that coincident pre and post-synaptic activity leads to an increase in processing capacity, or connection ‘strength’ between these respective neurons. The underlying cellular process is termed long term potentiation (LTP), meaning that the response of the post-synaptic cell is potentiated, as first shown in the hippocampus (Bliss and Collingridge, 1993). Through continuous coincident stimulation of different neuronal populations, it was observed that there was an increase in synaptic efficacy within the stimulated networks (Bliss and Collingridge, 1993).

The somatosensory system is an ideal candidate to study Hebbian-driven effects due to it being highly plastic (Simons and Land, 1987; Buonomano and Merzenich, 1998) in addition to its well characterised somatotopic map in both animal (Kaas et al., 1979) and human (Nelson and Chen, 2008). In animal models electrophysiological methods have shown that increasing the incidence of temporally correlated inputs to digits, either by fusing the digits (Clark et al., 1988) or training (Recanzone et al., 1992; Wang et al., 1995), results in increases in temporal coherence of distributed cortical responses and altered topography. Functional magnetic resonance imaging (fMRI) has also been used to show changes in topography following training and denervation (Pelled et al., 2007). These amongst other studies have shown significant cortical re-organisation and changes in synaptic temporal components after a relatively short period of time.

There have also been human studies encompassing behavioural measures (Godde et al., 1996) in conjunction with other measures such as fMRI (Pilz et al., 2004) and MEG (Godde et al., 2003) that observe similar changes. Human studies utilising the spatial resolution of fMRI have measured changes in both the location and size of the cortical representation (Pleger et al., 2001; Godde et al., 2003; Pleger et al., 2003; Hodzic et al., 2004) in response to continuous vibrotactile stimulation of a single digit, whilst synchronous stimulation (co-activation) of two digits results in their cortical representations moving closer together (Pilz et al., 2004). These changes are in good agreement with animal data (Jenkins et al., 1990), and have helped establish a working protocol that can be used in human studies.

Whilst great advancements have been made in understanding how LTP contributes to changes in synaptic interactions between neuronal groups, there are still questions that need to be addressed, namely (i) Are fMRI-derived functional connectivity measures sensitive to Hebbian-driven changes in the human brain?

ii) Can we relate these changes in connectivity to changes in cortical topography? In this study, the principle aim is to determine whether functional connectivity between digit representations within the primary somatosensory cortex (S1) is altered following co-activation of the digits in humans. We build on work by Pilz et al. (2004), using a similar co-activation paradigm, involving 3 hours of either synchronous or asynchronous co-activation of two digits on the right hand. In our study, fMRI was used to locate the two digit regions in S1 before and after co-activation. The BOLD time series was extracted from these regions and temporal coherence between the digit regions was determined. Subjects taking part in the synchronous paradigm were re-scanned after 3 weeks to determine if any changes had normalised. We hypothesise that functional connectivity between the co-activated digit representations will increase following synchronous co-activation due to increased synaptic excitability between the cortical regions, but not in the asynchronous case. The asynchronous control will enable us to determine the importance of the temporal coincidence of the inputs, and allow us to address the first key question of whether Hebbian-effects can be observed in the human brain. By relating these coherence changes to any shifts in the digit representations we will address the second aim concerning the underlying mechanisms of cortical reorganisation.

## Materials and methods

### Participants

This study was approved by the University of Liverpool ethical board and fully informed written consent was obtained from all subjects prior to participation. Thirty-one right handed healthy subjects were recruited from the student population. Participants were assigned to either the synchronous (n = 16) or asynchronous condition (n = 15). Two of the ‘asynchronous’ cohort did not complete both scanning sessions and so their data could not be used, leaving 29 subjects (17 females, 12 males; mean age: 25.6 ± 4.0 years). The study protocol consisted of a ‘pre’ baseline scan before the 3 hour co-activation followed by a ‘post-coactivation’ scan; all on the same day. Time between the end of the 3 hour co-activation and the start of the post coactivation scan protocol was minimised, and always less than 20 minutes.

We hypothesised that the synchronous condition would produce the greater brain changes, hence we invited the participants from the synchronous arm to return for a further scan after a period of 3 weeks to determine if any brain changes had normalised. This return scan was very much a secondary aim and due to practical constraints we could not scan all volunteers on this return visit. A total of ten participants returned and were scanned once on that day.

The same localiser paradigm (including amplitude and frequency of stimulation) and the same scan protocol were applied for all scan sessions and subjects.

### Co-activation paradigm

A vibrotactile stimulator, was used to stimulate digits 2 (D2) and 4 (D4) of the right hand. These tactile stimulators consist of a probe that vibrated by means of a piezoceramic wafer and carbon fibre encased in a ceramic box. Each digit had its own stimulator which was driven at a vibrational frequency of 30 Hz and the stimulation intensity was maintained for both co-activation and the fMRI localisation paradigm with an un-loaded peak to peak amplitude of 250 μm based on the voltage used to drive the piezoceramic wafer. The probe was placed on the middle of the fleshy tip part of each digit so that it would vibrate on the part with the most surface area of skin. The same person fixed the probe to the digit on each occasion to help ensure the same area was stimulated across sessions. Sinusoidal audio waveforms as defined in Presentation software (Neurobehavioural Systems, www.neurobs.com) were used to drive the stimulators. Digit co-activation was administered for 3 hours and consisted of brief stimulation pulses of 30 Hz for 50 ms duration (i.e. one and a half cycles, essentially a single ‘pulse’) with random and variable inter-stimulus intervals between 100 and 3000 ms, with a mean interval of 1000 ms. During synchronous co-activation, the pulses were delivered synchronously and in phase to each digit. For the asynchronous co-activation, a different pulse train was applied to each digit using pre-programmed waveforms such that pulses would rarely occur synchronously. Overall the number of stimuli was maintained to be the same for both the synchronous and asynchronous co-activation paradigms. In order to prevent subjects from attending to the stimulation during co-activation, subjects were encouraged to either read or watch a DVD.

### Localiser paradigm

**Fig. 1:**
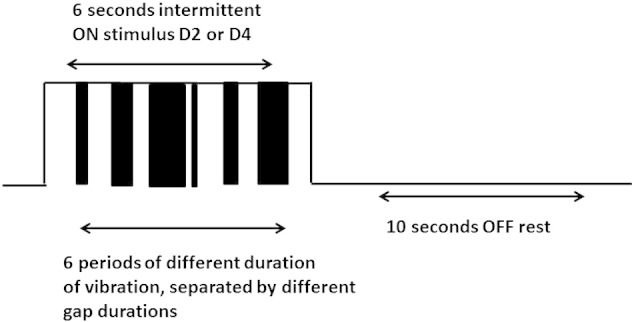
Stimulation paradigm for digit localisation.

The localiser paradigm was used during continuous fMRI to identify the cortical locations of the digits. This paradigm emulated designs from a previous study (Pilz et al., 2004), consisting of each stimulator vibrating at 30 Hz with an un-loaded peak to peak amplitude of 250 μm for an ON period of 6 seconds (with intermittent vibration of the stimulator during this period to reduce the effects of adaptation), and an OFF period of 10 seconds (Fig. 1). Intermittent vibrations were randomised with durations of 200, 300, 500, 750, 1200 and 1750 ms. Inter-vibrational gaps were also randomised with duration of 50, 100, 200, 400 and 550 ms. The paradigm consisted of 15 stimulations of each digit in a random order, lasting approximately 8 minutes. This was repeated once in each scanning session, giving a total of 30 stimulations of each digit. Whilst in the scanner, participants were instructed to respond on a button box using their left hand when they felt the vibrations begin on a new digit (i.e. the start of the 6 s ON period). This behavioural task was used to help maintain attention to the stimulation.

### Image acquisition

MR data was acquired using a Siemens 3 T Trio system (Siemens Medical Systems, Germany) and an 8-channel phased-array head coil for signal detection. A high resolution T_1_ weighted structural image with 1 mm isotropic voxels was collected using an MPRAGE pulse sequence.

fMRI BOLD data collected during the localiser task was derived from T_2_*-weighted EPI scans that were acquired with prospective motion correction with a TR of 2 s, TE of 35 ms, 2 mm slice thickness, 64 × 64 matrix and field of view of 128 mm giving 2 mm isotropic voxels. Coverage was limited to 28 slices with no gap, therefore EPI scans were planned on the high resolution T1-weighted structural images to ensure accurate coverage of the S1 region.

### Image analysis

#### Localisation of digit representations

All fMRI data analyses were carried out in BrainVoyager (Brain Innovations QX, The Netherlands). All data were analysed on an individual subject basis. Pre-processing consisted of slice-time correction, motion correction, temporal filtering (0.01 Hz high pass filter) and spatial smoothing (4 mm Gaussian kernel). In order to ascertain areas of relevant activity, data from both localiser runs were input into a general linear model for each individual. In order to separate activity relating to each digit, two regressors were defined to model the time course of stimulation applied to each digit (with box-car functions used to define each period of stimulation). This was convolved with a haemodynamic response function (a superposition of two gamma functions as is standard in BrainVoyager) to model the BOLD signal. Parameter estimates reflecting the amplitude of the BOLD response for each of these regressors were calculated at each voxel. A contrast image was then calculated, showing regions where the parameter estimate for one digit was significantly greater or lower than for the other digit. This contrast map thus depicted the location of each digit representation on the cortex.

High resolution T_1_ weighted structural images were acquired in both the pre and the post co-activation scan sessions. In order to compare digit localisations to previous work, the pre co-activation T_1_ weighted images were normalised to Talairach space. The post co-activation T_1_ weighted image was co-registered to the pre co-activation image and then normalised to Talairach space using the same transformation. Finally, the same transformation was applied to the co-registered BOLD contrast maps to bring them into Talairach space. Normalisation of structural images was performed using a combination of manual delineation (of key anatomical landmarks such as the anterior and posterior commissures) and automated linear transformation algorithms incorporated in Brainvoyager.

For each pre, post and return session, digit regions were defined following the approach described in a previous study (Vidyasagar and Parkes, 2011), which showed a high level of reproducibility across sessions. In brief, the t-statistic contrast maps were first thresholded to a significance level of p < 0.01, uncorrected, for visual inspection. A threshold of 200 voxels was set for identification of cortical activation sites for D2 and D4 based on findings from previous work (Vidyasagar and Parkes, 2011). Data were excluded from further analysis if they failed to show at least 200 contiguous voxels activated at this threshold for both digits. Activated volumes within the post-central gyrus, relating to individual digits were then further thresholded to the 200 most significant voxels. Within the thresholded regions the centre voxel coordinates were recorded.

The location of digit representations was recorded independently for each subject and each session (pre, post and return), and the Euclidean distance between digit representations was calculated. This approach can determine the distance between two digit representations with a 95% confidence interval of 1.7 mm (Vidyasagar and Parkes, 2011). Paired student *t*-tests were performed to determine whether individual digit locations and the distance between digits changed following synchronous and asynchronous stimulation.

#### Functional connectivity analysis

Functional connectivity was determined by calculating the temporal coherence between the fMRI time courses from the two digit regions. Coherence between neuronal groups has long been thought of as an effective and flexible mode of communication (Fries, 2005). We chose to measure coherence rather than correlation for two key reasons; coherence is a more sensitive measure of connection strength (Smith et al., 2011), and secondly, coherence allows separation of the stimulus-driven effects. As the digit stimulation occurs periodically (10 s OFF 6 s ON), this translates to a fixed frequency which can be separated from the ongoing activity.

In order to obtain data time courses from the same regions for both pre and post co-activation data, combined fMRI data from both the pre and post imaging sessions were used to define a single digit 2 (D2) and digit 4 (D4) region, using methods as described above. BOLD time-course data from both sessions were extracted from these regions. Using D2 and D4 regions defined separately from pre and post sessions would introduce a bias as, if the regions move together coherence will increase simply due to the increased sharing of a common vascular bed. When considering data from the return session, combined data from the pre and return sessions were used to define the two digit regions. In all cases, the BOLD time-course, without any temporal smoothing, was recorded from both digit regions (D2 and D4) for the localiser functional runs. Coherence between the time-course data from regions D2 and D4 was calculated using a fast Fourier transform algorithm implemented on Matlab version 2009a (http://www.mathworks.com) following the procedure described in (Sun et al., 2004). Welch's periodogram averaging method (as implemented in the Matlab function *mscohere*) was used to calculate the magnitude squared coherence between the frequencies of 0 and 0.15 Hz (in 20 steps of 0.0078 Hz).

Paired *t*-tests were used to determine differences in pre- and post- co-activation coherence at each frequency for both the synchronous and asynchronous case and for ‘pre’ compared to ‘return’, with significance defined as p < 0.0025 (reduced from 0.05 due to Bonferroni correction for the 20 tests performed). Low frequencies (< 0.1 Hz) have been shown to best reflect functional connectivity (Cordes et al., 2001), however, our localiser task frequency is 0.0625 Hz. Hence we calculated mean coherence for each condition between 0.01 and 0.1 Hz, ignoring the task frequency and the two frequencies on either side, in order to best reflect functional connectivity but avoid the task frequency. A 2-way ANOVA was performed using SPSS (IBM software, http://www-01.ibm.com/software/uk/analytics/spss/) with session (pre/post) as a within subject factor and intervention (synch/asynch) as a between subject factor in order to determine whether there was an interaction between session and intervention. This was performed for each of the output measures: coherence at the driving frequency, coherence at the non-driving frequencies and distance between the digits.

To determine the spatial specificity of the result we also extracted the BOLD time-course data from a separate control third region, ‘D_middle’. D_middle was a cube of size 216 voxels with centre coordinates exactly between the centre coordinates of the D2 and D4 regions, which were identified on an individual basis. Using the same approach as before, the coherence between D2 and D_middle and D4 and D_middle was calculated.

## Results

### Digit localisation

**Fig. 2:**
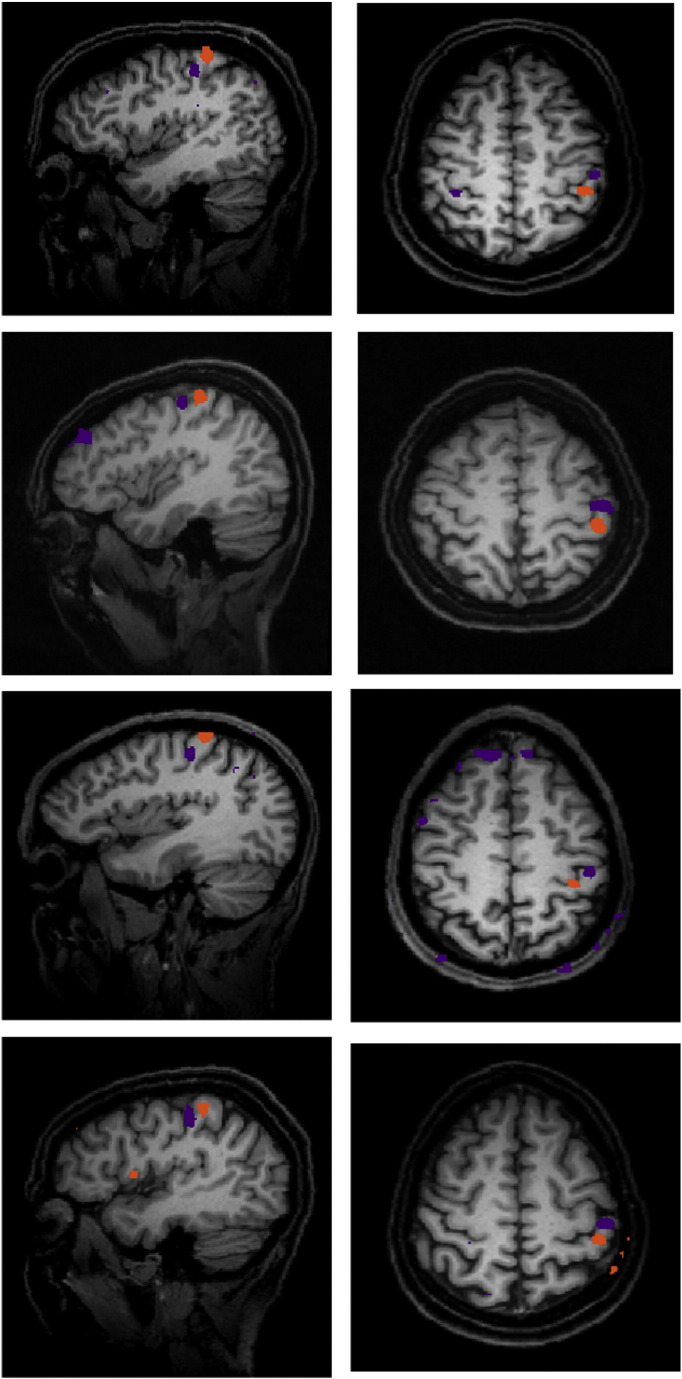
Location of digit representations on the cortical surface.

Fig. 2 shows examples from 4 typical subjects in sagittal and axial planes. The digit representations are localised to the posterior bank of the central sulcus, as was the case for all participants, identified as area 3b within S1. This is supported by the mean Talairach coordinates of (− 44 − 23 48) for D2 and (− 41 − 29 54) for D4 which are in good agreement with those identified as area 3b in previous work (Nelson and Chen, 2008). Another possible candidate area is area 1 within S1, which is also organised somatotopically, but is located in a more superior position on the crown of the postcentral gyrus and generally shows less robust activation than area 3b (Nelson and Chen, 2008). Further validation of the location of these areas was carried out using the SPM Anatomy toolbox (http://www.fil.ion.ucl.ac.uk/spm/ext/). For all except 2 subjects, both digit ROIs were identified to lie in area 3b. That is, of all sub-areas within S1, there was the highest probability that they were in area 3b. The two remaining subjects had one digit region (D2) in area 3b and the other in area 1, just on the periphery of area 3b. The digit localisation paradigm provided robust digit localisation in 24 out of the 29 participants for combined pre and post co-activation data. An area of 200 contiguous voxels was identified for D2 and D4 for each individual, based on previous work showing this size region to be highly reproducible (Vidyasagar and Parkes, 2011). Participants were excluded from subsequent analysis (3 participants from the synchronous and 2 participants from the asynchronous conditions, leaving 13 in the synchronous condition, including 10 returners, and 11 in the asynchronous condition) as they had failed to show 200 contiguously activated voxels at p < 0.01 uncorrected for both digits. It is unclear why some participants did not show clear digit activations and one can only speculate that it may be due to experimental error such as the digits were not in direct contact with the vibrating tactors and therefore they were not stimulated properly.

### Functional connectivity

**Fig. 3:**
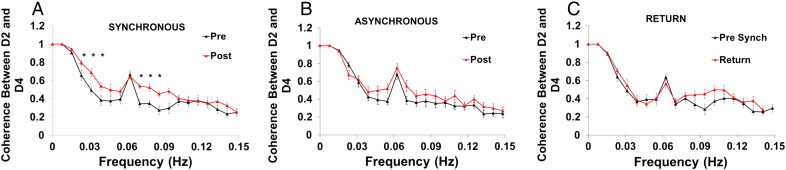
Coherence spectra during the localiser scan.

We extracted mean BOLD time-course data from the D2 and D4 cortical regions and computed the coherence between the data from these two regions on an individual basis. Fig. 3 shows the average coherence spectra across all participants during the localiser task for the synchronous (a), asynchronous (b) and return (c) sessions. It can be seen that coherence from the fMRI localiser activity between D2 and D4 significantly increases following the 3 hour synchronous co-activation paradigm over a range of frequencies from 0.02 to 0.09 Hz (Fig. 3A), but not in the asynchronous case (Fig. 3B). Comparing the spectra from the synchronous pre session and the return session 3 weeks later (Fig. 3C), it can be seen that the coherence profile in the return session has returned to the pre-co-activation level. Strong coherence is seen at the driving frequency of the task (0.0625 Hz), which is expected as both digit regions respond at the frequency of the task stimulus and so appear coherent. At frequencies away from the driving frequency it is possible that coherence is driven by spontaneous simultaneous neuronal activity between the two digit regions.

We thus compared the coherence between pre- and post- co-activation at i) the driving frequency of the vibrational stimulus and ii) the mean coherence at frequencies between 0.01 and 0.1 Hz but avoiding the driving frequency (Table 1). A 2 way ANOVA with session (pre/post) as a within subject factor and intervention (synchronous/asynchronous) as a between subject factor was carried out for the non driving frequencies. We found a main effect of session (F = 15.8, df = 1, p = 0.001). We found a significant interaction between session and intervention (F = 5.2, df = 1, p = 0.03) supporting the finding that coherence is specifically increased for the synchronous case. At the driving frequency, there was no effect of either intervention on coherence. This was confirmed using a similar ANOVA as described above, showing no significant effect of session (F = 0.5, df = 1, p = 0.5) or interaction between session and intervention (F = 1.3, df = 1, p = 0.3). Paired *t*-tests confirmed these findings, showing, at non-driving frequencies, coherence increases by 20% following synchronous co-activation (p = 0.0004, 2 tailed paired *t*-test), but was unchanged following asynchronous activation. No significant change is seen between the pre co-activation measurements and on return, indicating that the changes have normalised by 3 weeks.

Considering coherence change from the control site, D_middle, we found there to be no significant coherence change between D2 and D_middle, when considering either *t*-tests on the individual data-points or the mean coherence measure (average between 0.01 and 0.1 Hz but avoiding the driving frequency). Pre co-activation, mean coherence = 0.68 ± 0.03, post co-activation mean coherence = 0.72 ± 0.03, p = 0.1. For D4 and D_middle, there was no significant change when considering individual data-points, but we did find a significant increase in mean coherence: pre co-activation, mean coherence = 0.77 ± 0.02, post co-activation mean coherence = 0.85 ± 0.01, p = 0.004.

### Digit shifts

Previous work has considered the effect of co-activation on the cortical location of digit representations (Pilz et al., 2004), therefore we aimed to replicate these results. Table 2 shows the mean Euclidean distance between the two digit representations for the synchronous, asynchronous and return sessions, along with the paired *t*-test results of the pre and post comparisons for each condition. There was a trend to a reduction in distance between the cortical locations for digits 2 and 4 of 1.8 mm following synchronous co-activation (p = 0.09), with this difference normalising in the return condition. In the asynchronous condition however a significant (p = 0.02) average distance increase of 4.2 mm was observed. A 2 way ANOVA with session (pre/post) as a within subject factor and intervention (synchronous/asynchronous) as a between subject factor was carried out on the distance shifts. We did not find a main effect with session (F = 2.6, df = 1, p = 0.17), however a significant interaction between session and intervention was observed (F = 11.9, df = 1, p = 0.002) suggesting that these shifts were different for the synchronous/asynchronous conditions.

The Euclidean distance between the Talairach co-ordinates for D2 pre and post and D4 pre and post were calculated. We find that, for the synchronous case, the location of D4 changed significantly, moving to a more medial and inferior position towards D2 (Table 3). D2 did not move significantly.

### Relationship between coherence change and altered topography

**Fig. 4:**
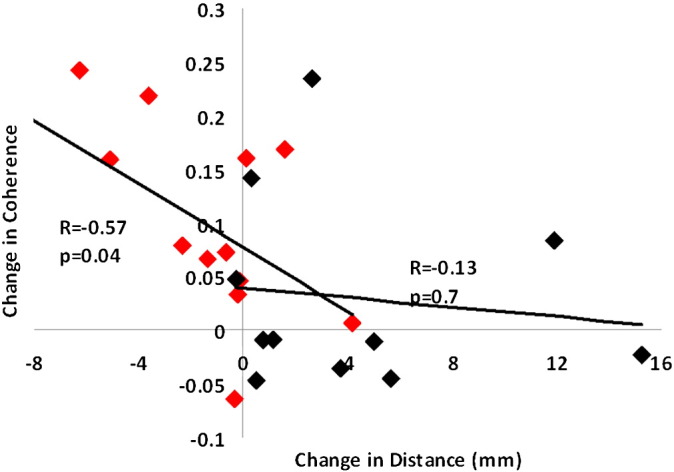
Relationship between coherence change and altered topography. Changes in coherence (y axis) are plotted against digit shifts (x axis) for the synchronous (red) and asynchronous (black) co-activation. Linear regression reveals a significant relationship between the two metrics for the synchronous case (R = − 0.57, p = 0.04), whilst the asynchronous has a low correlation that is not significant (R = − 0.13, p = 0.7).

If the coherence changes we see are related to the alterations in cortical topography, then we would expect a significant correlation between these two metrics across subjects. When considering changes across both synchronous and asynchronous co-activation, a significant relationship was found (r = − 0.47, p = 0.02, df = 22). Investigating this relationship in further detail Fig. 4 shows that this significance is primarily driven by the relationship between the synchronous co-activation paradigm and the related cortical shifts (r = − 0.57, p = 0.04, df = 11), whilst the asynchronous shows a relationship which is not significant (r = − 0.13,p = 0.7, df = 9).

## Discussion

This study focused on studying the underlying changes that occur in response to passive stimulation of the somatosensory system. Following continuous stimulation of digits 2 and 4 of the right hand over a 3 hour period we found significant changes in the temporal characteristics of the fMRI signal from these cortical regions in the form of a 20% increase in coherence. This increase in functional connectivity is driven specifically by the temporal pattern of the inputs. It occurs when inputs are synchronous but not when they are temporally unrelated (asynchronous). In other words, functional connectivity increases only after a period of coincident inputs to the two cortical regions. This suggests that Hebbian mechanisms are responsible for the observed change in functional connectivity. We found the coherence change to be spatially specific, as the coherence between D2 and the control region D_middle did not change following synchronous co-activation. Coherence between D4 and D_middle *did* change however, but we believe this is related to the shift in the D4 region following co-activation. We have shown that D4 moved significantly towards D2 following co-activation (whereas D2 remained stationary; Table 3), thus D4 will begin to encroach on the D_middle region, leading to increased coherence. The form of ‘training’ in this study differs from the learning paradigms as it is passive in nature, however studies in animal models have shown that similar passive stimulation has led to significant changes in both the spatial and temporal characteristics of the cortical system (Jenkins et al., 1990).

Temporal correlations and coherence of the BOLD signal has been shown to be one of the more sensitive methods of determining the level of interaction or ‘functional connectivity’ between different cortical regions (Sun et al., 2004; Smith et al., 2011). The direct relationship between BOLD coherence and oscillatory activity derived from electrophysiological measures are still unclear and difficult to ascertain due to differences in temporal resolution between the two methods. BOLD measures have been shown to be in line with LFP activity (Logothetis et al., 2001) and further resting state connectivity studies (Singh et al., 2002; Liu et al., 2010) have shown that the haemodynamic BOLD signal is closely related to the enveloped power band activity from electrophysiology measures. It is therefore possible that fMRI coherence still reflects neuronal coherence, but after passing through a number of broad vascular filters which essentially reduces the frequency of coherence. The paradigm used in this study consisted of regular blocks of the same activation which made it particularly suited for coherence analysis as the direct effect of the stimulation appears at a discrete frequency and can be studied in isolation of any changes in intrinsic connectivity.

In this study the largest changes in coherence were seen at frequencies below 0.1 Hz (Fig. 3), in agreement with the suggestion that this frequency range is most sensitive to neural connectivity (Cordes et al., 2001). It is noteworthy that 12 out of 13 subjects showed increased coherence following synchronous co-activation, highlighting the sensitivity of this measure to functional change. These changes could reflect an increase in synaptic weighting of the cortico-cortical connections between the two regions. This would cause increased coherence of intrinsic activity between the neurons in the two regions, leading to the increased coherence seen in the BOLD signal. This is supported by the fact that the change is only seen following synchronous co-activation and not asynchronous, showing that coincident input is required to bring about the change, something that is known to increase synaptic efficacy. There was no change in coherence at the driving frequency, suggesting that the observed coherence changes reflect alterations in intrinsic connectivity rather than being driven directly by the stimulus.

To our knowledge, this study is the first of its kind to focus on changes in the temporal characteristics of the BOLD signal between two cortical regions following a relatively short period of synchronous co-activation in humans. It is possible however to support the coherence changes observed in this study by looking at some of the behaviour measures carried out by previous groups (Godde et al., 2003; Pilz et al., 2004). For example, an increase in coherence between the two digits could explain the increased mislocalisation of stimuli on different digits seen by Kalisch et al. (Kalisch et al., 2007) and Pilz et al. (Pilz et al., 2004) following synchronous co-activation. If the co-activated neuronal groups now respond to each others stimulation (as evidenced by increased coherence) then localisation will be impaired.

In addition to the coherence changes, there was an observed trend in a change in distance between the two cortical sites following the synchronous and asynchronous co-activation protocols. A trend to decreased distance (Table 2) between the two regions suggests that the start of a topographical change in response to the synchronous co-activation is observed and is in agreement with observations in animals (Jenkins et al., 1990) and MEG findings from Godde et al. (Godde et al., 2003) as well as fMRI measures by Pilz et al. (Pilz et al., 2004). The asynchronous co-activation resulted in a significant increase in separation distance between the two cortical regions (Table 2). Again this finding is in agreement with previous work (Pilz et al., 2004), whereby only adjacent digits were used, whereas we have used slightly more distant digits that show that this effect is still apparent for more distant cortical representations. Additionally, a number of early studies in non-human primates support our findings, showing merging of cortical representations due to increased temporal correlation of sensory inputs following digit syndactyly (Clark et al., 1988; Allard et al., 1991).

The main mechanism suggested to underlie this changing topography is again strengthening of intra-cortical connections via a Hebbian learning rule (Grajski and Merzenich, 1990; Pilz et al., 2004). Support for this comes from earlier studies exploring the mechanisms of plasticity induced by co-activation of a single digit. For example, application of an NMDA-receptor blocker prevents enlargement of the cortical response to co-activation on a single digit (Dinse et al., 2003). In the current study, a significant relationship is observed between the coherence changes and the spatial shifts (Fig. 4) for the synchronous case. This association supports the notion that LTP-driven alteration in synaptic efficacy leads to both altered spatial topography and altered temporal coherence (Eyding et al., 2002; Narayanan and Johnston, 2007). Following asynchronous co-activation, we observed increased digit separation but no reduction in coherence, and indeed no correlation between coherence change and spatial shifts (Fig. 4), suggesting a different underlying mechanism in this case. In addition to the reduced distance between the two digits following co-activation, a separate analysis of the changes in location for individual digits before and after coactivation was shown to be interesting. A significant shift in the locations for D4 following only the synchronous case was observed following co-activation. It is difficult to be certain why D4 solely shifts, but one possible explanation may be that this is related to the increased general use of D2 making it more fixed in its cortical position.

There were some limitations to this study due to technical and practical constraints. It would have been advantageous to obtain full brain coverage from all participants in order to investigate any potential changes occurring at the thalamocortical level. However, we believe this study has established a sensitive measure of cortical processing changes in response to a Hebbian-like stimulation. This may be a starting point to establish a means of measuring LTP related changes in the human brain in vivo, with important potential applications.

## Figures and Tables

**Table 1:** Coherence measures from functional task.

	Synchronous		Asynchronous		Return	
	Driving frequency	Other frequencies	Driving frequency	Other frequencies	Driving frequency	Other frequencies
Pre	0.66 ± 0.04	0.56 ± 0.03	0.68 ± 0.06	0.60 ± 0.02	0.62 ± 0.03	0.57 ± 0.04
Post (or return)	0.65 ± 0.04	0.67 ± 0.02	0.75 ± 0.05	0.63 ± 0.02	0.60 ± 0.06	0.61 ± 0.03
p-Value	0.8	0.0004	0.09	0.3	0.7	0.2

Coherence measurements during the digit localiser paradigm (mean ± SE).

**Table 2:** Cortical shifts for D2 and D4.

	Synchronous	Asynchronous	Return
Pre	12.0 ± 0.5	12.8 ± 1.0	11.6 ± 1.0
Post (or return)	10.2 ± 0.8	17.0 ± 1.6	11.0 ± 1.1
p-Value (2 tailed paired *t*-test)	0.09	0.02	0.3

Distance (mm) between cortical representations of D2 and D4 (mean ± SE).

**Table 3:** Average locations of digits on the cortical surface in Talairach space pre and post synchronous or asynchronous coactivation.

	Pre (mm, mean ± SD)			Post (mm, mean ± SD)			Significance (p value)		
	x	y	z	x	y	z	x	y	z
Synchronous									
D2	− 43.1 ± 3.5	− 23.0 ± 3.4	49.0 ± 3.4	− 44.4 ± 4.0	− 22.7 ± 4.5	47.8 ± 4.1	0.4	0.4	0.3
D4	− 38.8 ± 3.3	− 30.2 ± 4.2	55.8 ± 2.4	− 42.0 ± 7.3	− 28.3 ± 4.9	51.2 ± 6.0	0.04*	0.06	0.01*
Asynchronous									
D2	− 42.5 ± 5.2	− 22.3 ± 3.7	49.9 ± 4.5	− 43.9 ± 5.5	− 22.6 ± 8.8	46.4 ± 6.2	0.5	0.9	0.06
D4	− 41.7 ± 3.9	− 29.3 ± 6.8	54.7 ± 2.2	− 40.6 ± 6.6	− 27.0 ± 7.5	54.6 ± 8.3	0.2	0.3	1.0

Asterisked values represent planes with significant (p < 0.05) shifts following the 3 hour co-activation.
